# Early-Life Cognitive Activity Is Related to Reduced Neurodegeneration in Alzheimer Signature Regions in Late Life

**DOI:** 10.3389/fnagi.2018.00070

**Published:** 2018-03-22

**Authors:** Kang Ko, Min Soo Byun, Dahyun Yi, Jun Ho Lee, Chan Hyung Kim, Dong Young Lee

**Affiliations:** ^1^Department of Neuropsychiatry, Seoul National University Hospital, Seoul, South Korea; ^2^Department of Psychiatry, Yonsei University College of Medicine, Seoul, South Korea; ^3^Institute of Human Behavioral Medicine, Medical Research Center, Seoul National University, Seoul, South Korea; ^4^Department of Psychiatry, Seoul National University College of Medicine, Seoul, South Korea; ^5^Institute of Behavioral Science in Medicine, Yonsei University College of Medicine, Seoul, South Korea

**Keywords:** cognitive activity, early life, midlife, late life, Alzheimer’s disease, neurodegeneration, amyloid beta deposition, the KBASE study

## Abstract

**Background:** Although increased cognitive activity (CA), both current and past, is known to be associated with a decreased occurrence of Alzheimer’s disease (AD) dementia in older adults, the exact neural mechanisms underlying the association between CA during different stages of life and human dementia remain unclear. Therefore, we investigated whether CA during different life stages is associated with cerebral amyloid-beta (Aβ) pathology and AD-related neurodegeneration in non-demented older adults.

**Methods:** Cross-sectional analyses of data collected between April 2014 and March 2016 from the Korean Brain Aging Study for Early Diagnosis and Prediction of Alzheimer’s Disease (KBASE), an ongoing prospective cohort. In total, 321 community-dwelling, non-demented older adults were involved in this study. Cerebral Aβ deposition and Aβ positivity were measured using ^11^C-Pittsburgh compound B (PiB)-positron emission tomography (PET). AD-signature region cerebral glucose metabolism (AD-CMglu) and AD-signature region neurodegeneration (AD-ND) positivity were measured using ^18^F-fluorodeoxyglucose (FDG)-PET. In addition, CA in early, mid, and late life was systematically evaluated using a structured questionnaire.

**Results:** Of the 321 participants, 254 were cognitively normal (CN) and 67 had mild cognitive impairment (MCI). The mean age of participants was 69.6 years old [standard deviation (SD) = 8.0]. Higher early-life CA (CA_early_) was associated with significantly increased AD-CMglu (*B* = 0.035, SE = 0.013, *P* = 0.009) and a decreasing trend of AD-ND positivity (OR = 0.65, 95% CI 0.43–0.98, *P* = 0.04) but was not associated with Aβ deposition or positivity. We observed no association between midlife CA (CA_mid_) and any AD-related brain changes. Late-life CA (CA_late_) showed an association with both global Aβ deposition and AD-CMglu, although it was not statistically significant. Sensitivity analyses controlling for current depression or conducted only for CN individuals revealed similar results.

**Conclusion:** Our results suggest that CA in early life may be protective against late-life AD-related neurodegeneration, independently of cerebral Aβ pathology.

## Introduction

Increased cognitive activity (CA), both current and past, is known to be associated with reduced cognitive decline (Marquine et al., 2012; Wilson et al., 2012, 2013; Hughes et al., 2015; Arfanakis et al., 2016) and the occurrence of Alzheimer’s disease (AD) dementia (Wilson et al., 2002a, 2007; Sattler et al., 2012) in the elderly. However, the exact pathological process underlying this inverse association between CA and AD dementia remains unclear.

To explore the pathological process, several studies investigated the association between the degree of CA and both cerebral amyloid-beta (Aβ) pathology (Landau et al., 2012; Vemuri et al., 2012, 2016, 2017; Wirth et al., 2014; Gidicsin et al., 2015) and neurodegeneration (Valenzuela et al., 2008; Vemuri et al., 2012, 2016, 2017; Gidicsin et al., 2015) using *in vivo* AD neuroimaging biomarkers. The results from these studies are, however, controversial. One possible explanation for this controversy is that the brain has different physiological or pathological properties during different stages of life. The influence of a certain life experience, such as CA, on the brain may vary at different stages of life. Nevertheless, most previous studies exploring the association between CA and AD biomarkers did not take into account different life stages and simply classified all CA into simple categories, mainly current or past (Landau et al., 2012; Vemuri et al., 2012; Gidicsin et al., 2015), or focused only on either midlife CA (CA_mid_) (Vemuri et al., 2016, 2017) or late-life CA (CA_late_) (Valenzuela et al., 2008).

Early life (i.e., childhood and early adulthood) is a critical period for brain development characterized by neural plasticity (Chugani et al., 1987; Andersen, 2003; Dekhtyar et al., 2016). Previous studies have shown that early-life CA (CA_early_) is associated with reduced late-life cognitive decline and progression to cognitive disorders in later life (Wilson et al., 2013, 2015; Dekhtyar et al., 2016), suggesting that CA_early_ is closely related to increases in cognitive reserve (CR). CR refers to functional rather than structural or quantitative aspects of the brain, and may explain why some people are more resilient to cognitive decline than others who present with the same level of pathology (Stern, 2012). In contrast, CA in mid or late life stages is less beneficial to individuals, given that brain plasticity is limited during mid- and late life (Leuner et al., 2007; Kolb and Gibb, 2011).

The accumulation of cerebral Aβ pathology begins 10–20 years prior to AD dementia (Villemagne et al., 2013) and its prevalence in non-demented persons typically increases from mid- to late life (Jansen et al., 2015). Thus, cerebral Aβ pathology is rarely observed in the early-life period. Therefore, it is more reasonable to assume that CA or other brain affecting activities may influence the occurrence of Aβ pathology when they are applied in mid or late life rather than in early life. Some studies have reported an association between CA_mid_ and Aβ deposition (Wirth et al., 2014; Vemuri et al., 2016). In the case of late life, however, about half of the cognitively healthy elderly already have amyloid or neurodegeneration abnormalities and the estimated frequency of normal AD biomarker status decreases continuously with age (Jack et al., 2014). Therefore, the accumulation of amyloid and/or neurodegeneration itself might reduce participation in CA in late life, although a few studies have reported a beneficial effect of congitive training or exercise in late life on brain function as well as cognive performance (Snowball et al., 2013; Shah et al., 2014; Lampit et al., 2015).

We hypothesized that CA during different stages of life is differentially associated with cerebral Aβ pathology and AD-related neurodegeneration in non-demented older adults. More specifically, we formulated three working hypotheses. First, CA_early_ is inversely associated with the degree of AD-related neurodegeneration, including neuronal or synaptic dysfunction in late life. Second, CA_mid_ is inversely associated with cerebral Aβ pathology in late life. Third, CA_late_ is inversely associated with both cerebral Aβ pathology and AD-related neurodegeneration in late life.

To test our hypotheses, we measured cerebral Aβ pathology using ^11^C-Pittsburgh compound B (PiB)-positron emission tomography (PET) and AD-related neurodegeneration using ^18^F-fluorodeoxyglucose (FDG)-PET. We selected cerebral glucose metabolism (CMglu) on FDG-PET as a neurodegeneration marker because it is a reliable index of regional neuronal or synaptic function (Sokoloff, 1981; Jueptner and Weiller, 1995), and specific regional hypometabolism in the temporo-parietal cortices is a reliable and sensitive measure of AD-related neurodegeneration, which appears earlier than structural brain changes on magnetic resonance imaging (MRI) (Jack et al., 2014, 2015, 2016). CA in early, mid, and late life was assessed using a structured questionnaire (Wilson et al., 2005, 2007; Barnes et al., 2006). We further investigated the moderating effects of apolipoprotein E ε4 (*APOE4*) on the relationship between CA and AD-related brain changes, as CA is particularly protective in *APOE4* carriers for the risk of dementia onset (Carlson et al., 2008) and Aβ accumulation (Wirth et al., 2014; Vemuri et al., 2016).

## Materials and Methods

### Participants

This study was part of the Korean Brain Aging Study for Early Diagnosis and Prediction of Alzheimer’s Disease (KBASE), an ongoing prospective cohort study, which began in 2014 and was designed to identify novel biomarkers for AD and explore various lifetime experiences contributing to AD-related brain changes. The current study included 321 community-dwelling elderly individuals without dementia who were at least 55 years old and enrolled between April 2014 and March 2016.

The study participants consisted of 254 cognitively normal (CN) and 67 subjects with mild cognitive impairment (MCI). All individuals with MCI met the current consensus criteria for amnestic MCI: (1) memory complaints confirmed by an informant; (2) objective memory impairment, (3) preserved global cognitive function; (4) independence in functional activities; and (5) no dementia. All MCI individuals had a global clinical dementia rating (CDR) of 0.5. In terms of Criterion 2, the age-, education-, and gender-adjusted z-scores for at least 1 of the 4 episodic memory tests was less than -1.0. The four memory tests included Word List Memory, Word List Recall, Word List Recognition, and Constructional Recall tests, which are included in the Korean version of the Consortium to Establish a Registry for Alzheimer’s Disease (CERAD-K) neuropsychological battery. The CN group consisted of participants with a global CDR of 0 and lack of an MCI or dementia diagnosis. The exclusion criteria were current serious medical, psychiatric, or neurological disorders that may influence mental functioning; the presence of severe communication problems that would hinder the clinical interview or brain imaging process; *in vivo* devices or a mental status that prevented us from performing the brain MRI; absence of a reliable informant; illiteracy; participation in a different clinical trial; and treatment with an investigational product. The Institutional Review Board of the Seoul National University Hospital and Seoul Metropolitan Government-Seoul National University Boramae Medical Center, South Korea, approved this study, and subjects and their legal representatives provided written consent.

### Clinical Assessment

All participants received standardized clinical assessments by trained psychiatrists based on the KBASE clinical assessment protocol, which incorporated the CERAD-K (Lee et al., 2002). KBASE neuropsychological assessments incorporating the CERAD-K neuropsychological battery (Lee et al., 2004) were also administered to all participants by trained neuropsychologists. Genomic DNA was extracted from whole blood and apolipoprotein E (*APOE)* genotyping was performed as described previously (Wenham et al., 1991). *APOE4* carrier status was considered positive if the participant had at least one *APOE4* allele.

### Assessment of Early-, Mid-, and Late-Life CA

Participant CA was assessed using a 39-item expanded version (Wilson et al., 2005, 2007; Barnes et al., 2006) of a previously reported 25-item autobiographical questionnaire (Wilson et al., 2003; Landau et al., 2012), which was shown to have sufficient internal consistency and temporal stability. Items included relatively common activities with few barriers to participation, such as reading newspapers, magazines, or books; visiting a museum or library; attending a concert, play, or musical; writing letters; and playing games. Individuals completed the questionnaire at a baseline evaluation point. Frequency of participation was rated from 1 (once a year or less) to 5 (daily or approximately daily). There were 9 current (i.e., late life) activities and 30 previous activities including 11 related to childhood (6–12 years of age), 10 related to young adulthood (18 years of age), and 9 related to midlife (40 years of age). Item scores were averaged to yield separate values for each age period. The CA_early_ score was determined by averaging childhood and young adulthood scores.

### PiB-PET Acquisition and Processing

Participants underwent simultaneous three-dimensional (3D) PiB-PET and 3D T1-weighted MRI using a 3.0T Biograph mMR (PET-MR) scanner (Siemens, Washington, DC, United States) according to the manufacturer’s protocols. Details of PiB-PET imaging acquisition and preprocessing are described elsewhere (Supplementary Material).

The automatic anatomic labeling algorithm and a region combining method (Reiman et al., 2009) were conducted to determine regions of interest (ROIs) and to characterize the PiB retention level in the frontal, lateral parietal, posterior cingulate-precuneus, and lateral temporal regions. The standardized uptake value ratios (SUVRs) were calculated by dividing the mean value for all voxels within each ROI by the mean cerebellar uptake value in the same image. Each participant was classified as cerebral Aβ positive if the SUVR value was >1.4. A global cortical ROI consisting of the four ROIs was defined, and a global Aβ deposition value was generated by dividing the mean value for all voxels of the global cortical ROI by the mean cerebellar uptake value in the same image (Choe et al., 2014).

### FDG-PET Acquisition and Processing

Participants also underwent FDG-PET imaging using the same PET-MR machine, as described above. Details of FDG-PET image acquisition and preprocessing are described in the Supplementary Material. AD-signature FDG ROIs including the angular gyri, posterior cingulate cortex, and inferior temporal gyri, which are known to be sensitive to changes associated with AD (Jack et al., 2014, 2015) were determined. AD-signature region cerebral glucose metabolism (AD-CMglu) was defined as a voxel-weighted mean SUVR extracted from the AD-signature FDG ROIs, and AD-signature region neurodegeneration (AD-ND) positivity was defined as AD-CMglu <1.386. Detailed methods used to define the threshold for abnormality of each neurodegeneration biomarker are described in the Supplementary Material.

### Statistical Analysis

The associations between CA (independent variable) at each life stage and global Aβ deposition or AD-CMglu (dependent variables) were examined using multiple linear regression analyses controlling for age, sex, years of education, and *APOE4* carrier status as covariates. Multiple logistic regression analyses were conducted to test the association between CA at each life stage (independent variable) and Aβ or AD-ND positivity (independent variables). In this analysis, we also controlled for age, sex, years of education, and *APOE4* carrier status. Sensitivity analyses were conducted using the same analyses, but included only CN subjects to exclude the possibility of recall bias due to MCI. We also performed the same analyses but additionally controlled for geriatric depression using the Geriatric Depression Scale (GDS) (Yesavage et al., 1983), since current depression may influence CA and brain state. We set a *P*-value less than 0.0167 (=0.05/3) as the threshold for statistical significance, given that CA during the three life stages (i.e., CA_early_, CA_mid_, and CA_late_) were explored for AD-related brain changes. In the event that CA significantly influences Aβ-related brain changes, we further explored the moderating effects of *APOE4* using a generalized linear model analysis, including a CA × *APOE4* interaction term, as well as CA and *APOE4* as independent variables, controlling for age, sex, and education as covariates. In this case, a *P*-value less than 0.05 was indicative of statistical significance. All statistical analyses were conducted using SPSS Statistics version 23.0 (IBM Corp., Armonk, NY, United States).

## Results

The characteristics of the study participants are shown in **Table 1**. Both global Aβ deposition and AD-CMglu were weakly correlated with clinical variables. Global Aβ deposition was inversely associated with CERAD total score (Kendall’s tau = -0.19, *p* < 0.001) and was positively associated with CDR sum of boxes (Kendall’s tau = 0.33, *p* < 0.001). AD-CMglu showed a similar association with CERAD total score (Kendall’s tau = 0.16, *p* < 0.001) and was inversely associated with CDR-SOB (Kendall’s tau = -0.26, *p* < 0.001). Global Aβ deposition and AD-CMglu were weakly correlated with each other (Kendall’s tau = -0.17, *P* < 0.001). CA_early_ was moderately correlated with CA_mid_ (Kendall’s tau = 0.52, *P* < 0.001) and CA_late_ (Kendall’s tau = 0.43, *P* < 0.001). CA_mid_ and CA_late_ were also moderately correlated (Kendall’s tau = 0.51, *P* < 0.001).

**Table 1:** Participant characteristics.

Characteristics	All participants
No. of study participants	321
Age, years	69.60 (7.99)
No. of females (%)	180 (56.07)
No. of *ApoE4* carriers (%)^a^	79 (24.61)
Education, year	11.54 (4.69)
MMSE score (maximum, 30)	25.92 (3.35)
GDS score (maximum, 30)	5.53 (5.33)
MCI, no. (%)	67 (20.87)
CA score (maximum, 5)	
Early life	2.24 (0.69)
Midlife	2.37 (0.82)
Late life	2.57 (0.73)
Global cerebral Aβ deposition, SUVR	1.28 (0.37)
Aβ positivity (%)	68 (21.18)
AD-CMglu, SUVR	1.39 (0.13)
AD-ND positivity (%)	151 (47.04)

*APOE4, apolipoprotein E ε4; MMSE, Mini-Mental State Examination; GDS, Geriatric Depression Scale; MCI, mild cognitive impairment; CA, cognitive activity; Aβ, amyloid-beta; SUVR, standardized uptake value ratio; AD-CMglu, Alzheimer’s disease signature region cerebral glucose metabolism; AD-ND, AD-signature region neurodegeneration. Data are presented as mean (SD) unless otherwise indicated. ^*a*^ApoE4 carriers are the percentage of individuals with at least one APOE4 allele*.

### Early-Life CA and AD-Related Brain Changes

**FIGURE 1:**
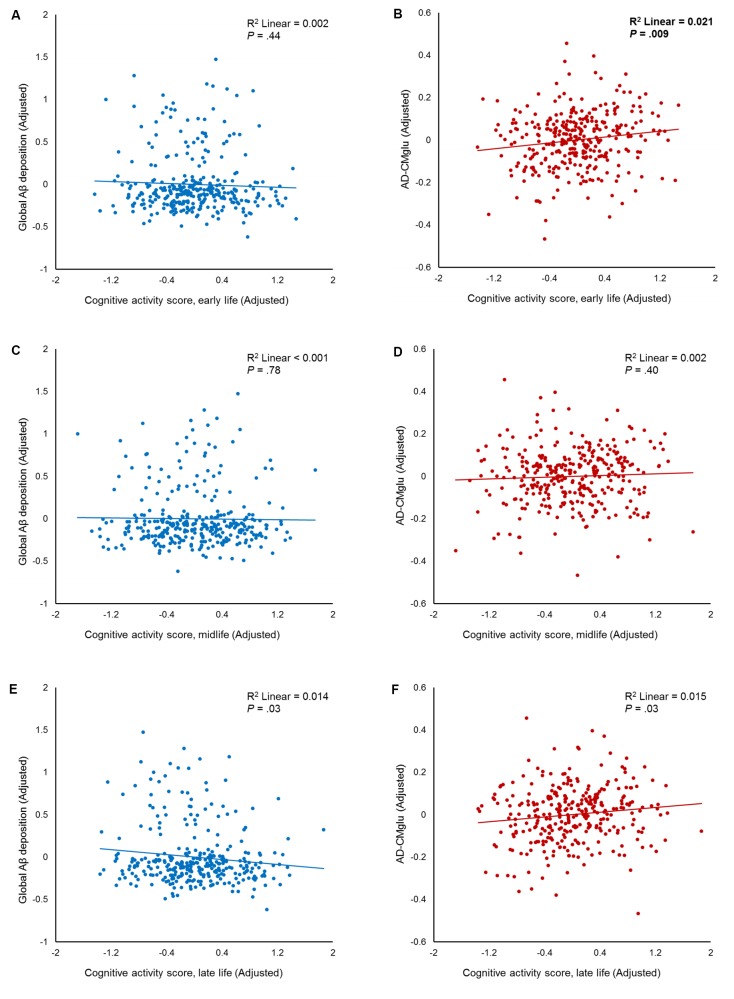
Partial regression plots showing the effect of participating in cognitive activity (CA) during each life period. The results of the independent multiple linear regression model with age, gender, education, and apolipoprotein E ε4 as covariates are presented. **(A,B)** Plots for early-life CA (CA_early)_ and amyloid-beta (Aβ), as well as for Alzheimer’s disease signature region cerebral glucose metabolism (AD-CMglu). A significant association was observed between CA_early_ and AD-CMglu, but not Aβ. **(C,D)** Plots for midlife CA (CA_mid)_ and Aβ, as well as for AD-CMglu. **(E,F)** Plots for late-life CA (CA_late)_ and Aβ, as well as for AD-CMglu.

We observed no association between CA_early_ and global Aβ deposition (**Figure 1A** and **Table 2**). Similarly, no significant association between CA_early_ and Aβ positivity was observed (**Table 3**). In contrast, there was a significant positive association between CA_early_ and AD-CMglu (**Figure 1B** and **Table 2**). We observed a trend for a negative association between CA_early_ and AD-ND positivity, although this was not statistically significant (**Table 3**). We explored moderation effects of *APOE4* on the association between CA_early_ and AD-CMglu, which showed a statistically significant result in the main effect analysis. We observed no CA_early_ × *APOE4* interaction on AD-CMglu (Supplementary Table e-1).

**Table 2:** Association between cognitive activities (CAs) in each life period and global cerebral amyloid-beta (Aβ) deposition and Alzheimer’s disease signature region cerebral glucose metabolism (AD-CMglu).

	B	SE	Beta	*P*	*P*_B_
**Global Aβ deposition**					
Early life	-0.028	0.036	-0.052	0.44	>0.99
Midlife	-0.009	0.032	-0.020	0.78	>0.99
Late life	-0.072	0.033	-0.142	0.03	0.10
**AD-CMglu**					
Early life	0.035	0.013	0.180	0.009	0.03
Midlife	0.010	0.012	0.061	0.40	>0.99
Late life	0.028	0.013	0.150	0.03	0.08

*Aβ, amyloid-beta; AD-CMglu, Alzheimer’s disease signature region cerebral glucose metabolism. The results of the independent multiple linear regression model with age, gender, education, and apolipoprotein E ε4 as covariates are presented. P_*B*_: P-value corrected by Bonferroni’s method*.

**Table 3:** Association between cognitive activities in each life period and Aβ and AD-signature region neurodegeneration (AD-ND) positivity.

	Adjusted OR	95% CI	*P*	*P*_B_
**Aβ positivity**				
Early life	0.840	0.500–1.410	0.51	>0.99
Midlife	0.939	0.594–1.482	0.79	>0.99
Late life	0.786	0.484–1.277	0.33	>0.99
**AD-ND positivity**				
Early life	0.648	0.427–0.983	0.04	0.13
Midlife	0.790	0.550–1.134	0.20	0.60
Late life	0.712	0.483–1.050	0.09	0.26

*OR, odds ratio; CI, confidence interval; Aβ, amyloid-beta; AD-ND, Alzheimer’s disease signature region neurodegeneration. The results of the independent multiple logistic regression model with age, gender, education, and apolipoprotein E ε4 as covariates are presented. P_*B*_: P-value corrected by Bonferroni’s method*.

### Midlife CA and AD-Related Brain Changes

We observed no association between CA_mid_ and global Aβ deposition or AD-CMglu (**Figures 1C,D** and **Table 2**). CA_mid_ was also not associated with Aβ or AD-ND positivity (**Table 3**).

### Late-Life CA and AD-Related Brain Changes

We observed a trend-level association between CA_late_ and both global Aβ deposition and AD-CMglu, although this association was not significant (**Figures 1E,F** and **Table 2**). CA_late_ was not associated with Aβ or AD-ND positivity (**Table 3**).

### Sensitivity Analysis

Even when the GDS was additionally controlled for age, education, gender, and *APOE4*, the results from the multiple linear or logistic regression analyses were similar (Supplementary Tables e-2, e-3). When the same analyses were conducted for the CN subgroup only, CA_early_ showed trend-level associations with AD-CMglu and AD-ND positivity (Supplementary Tables e-4, e-5), although the association was not statistically significant. We observed no association between CA_mid_ or CA_late_ and any AD-related brain changes. Moreover, because CA_early_ was correlated with CA_mid_ and CA_late_, we controlled for CA_early_ in addition to age, sex, education, and *APOE4* when analyzing the relationship of CA_mid_ or CA_late_ to AD-related brain changes. As shown in the Supplementary Tables e-6, e-7, the results were almost the same, even after controlling for the effects of CA_early_.

## Discussion

The results of this study generally support the hypothesis that CA during different life stages is differentially associated with cerebral Aβ pathology and AD-related neurodegeneration in non-demented older adults. With regard to the three working hypotheses, our findings supported the first hypothesis: CA_early_ was inversely associated with the degree of AD-related neurodegeneration in late life. In contrast, we could not accept the second hypothesis (i.e., an inverse association between CA_mid_ and cerebral Aβ pathology in late life) or the third (i.e., a significant inverse association between CA_late_ and both cerebral Aβ pathology and AD-related neurodegeneration in late life).

Our study is the first to verify the association between CA_early_ and AD-CMglu in late life, suggesting the presence of a potential mechanism underlying the inverse association between CA_early_ and AD dementia or cognitive decline. Previous studies have reported that childhood CA could reduce cognitive decline (Wilson et al., 2013) and music or foreign language training in early life was associated with a lower risk of MCI or AD dementia (Wilson et al., 2015). Another study showed that a complex occupation could not compensate for low school grades at a young age to prevent dementia, suggesting that early life is a critical period for increasing CR against dementia (Dekhtyar et al., 2016). To the best of our knowledge, no previous human studies have focused on the direct relationship between CA_early_ and brain changes in late life.

The association between CA_early_ and AD-CMglu in late life may be explained by the influence of CA_early_ on brain developmental processes (Chugani et al., 1987; Benes et al., 1994; Paus et al., 1999; Andersen, 2003), such as synaptogenesis and pruning during the early-life period in particular (Tau and Peterson, 2010). As activity-dependent mechanisms could modulate these processes, especially in early life (Bourgeois et al., 1989; Goodman and Shatz, 1993; Hata and Stryker, 1994; Kleim et al., 1996; Baker et al., 2017), it may be that CA_early_ promotes synaptogenesis and/or pruning in humans offers a plausible explanation. Metabolic changes measured by FDG-PET may reflect energy expenditures of these processes (Chugani et al., 1987). Other animal studies also suggest that early-life cognitive enrichment has various protective effects on the brain by increasing neurotrophic factors (Wolf et al., 2006) or gene/protein expression related to synaptic plasticity (Costa et al., 2007). However, the influence of common genetic predisposition cannot be completely excluded when addressing the association between CA_early_ and neurodegeneration in late life. A certain genetic factor may be related to both more CA participation in early life and less neurodegeneration in late life (Fox et al., 2010).

Educational level is associated with the level of CA, regardless of life period (Wilson et al., 2002b, 2013; Barnes et al., 2006; Gidicsin et al., 2015). Our data also show a similar association between years of education and CA_early_ (Kendall’s tau = 0.43, *P* < 0.001), CA_mid_ (Kendall’s tau = 0.48, *P* < 0.001), and CA_late_ (Kendall’s tau = 0.45, *P* < 0.001). A previous report showed that higher-level education, related to early-life enrichment, was associated with reduced age-related alterations of cerebrospinal fluid (CSF) neurodegeneration biomarkers (e.g., CSF total-tau, phosphorylated-tau), but not with amyloid biomarkers (CSF Aβ) (Almeida et al., 2015), similar to our observation for CA_early,_ Aβ pathology, and neurodegeneration. Nevertheless, because the aim of this study was to investigate the differential effect of CA during different life stages on *in vivo* AD pathology, we applied a lifetime CA questionnaire instead of simply using years of education as a measure of CA. In the current study, CA_early_ had a significant inverse relationship with AD-related neurodegeneration, while CA_mid_ and CA_late_ did not, after controlling for the level of education. This finding suggests that CA_early_ itself is potentially protective against late-life neurodegeneration or related cognitive decline, regardless of educational attainment.

An exploratory analysis to investigate the moderating effects of *APOE4* revealed no significant interaction between CA_early_ and *APOE4* on AD-CMglu. This finding may be explained by previous reports indicating that *APOE4*-related cognitive changes generally occur during mid or late life, as opposed to early life (Ruiz et al., 2010; Richter-Schmidinger et al., 2011; Wisdom et al., 2011). A previous meta-analysis of 20 studies also demonstrated that *APOE4* was not associated with cognitive function in young adults, adolescents, or children (Ihle et al., 2012).

Midlife CA was not associated with late-life Aβ deposition, which did not support our second hypothesis. Similar to our current finding, Mayo investigators reported no association between CA_mid_ and late-life Aβ deposition, in general, in non-demented elderly (Vemuri et al., 2016, 2017). They also showed that high CA_mid_ was associated with lower Aβ deposition in highly educated *APOE4* carriers (Vemuri et al., 2016). They proposed that a reverse causality may explain their finding: among highly educated *APOE4* carriers, those with higher Aβ deposition in middle age are most likely to experience subtle cognitive symptoms at that time and, consequently, avoid intellectual activity (Vemuri et al., 2016). We conducted similar analyses for highly educated (>14 years) *APOE4* carriers, but did not find any significant associations between CA_mid_ and Aβ deposition. Such discrepancies may be associated with the time frame for CA_mid_. We defined CA_mid_ as CA at the age of 40 years, while Mayo investigators measured CA_mid_ at 50–65 years of age. Younger individuals are less likely to be influenced by the reverse causality issue. With respect to neurodegeneration, no association between CA_mid_ and AD-CMglu or AD-ND positivity was observed, which is consistent with previous reports (Vemuri et al., 2016, 2017).

Although not statistically significant, CA_late_ showed a trend association with global Aβ deposition and AD-CMglu. This may be explained by reverse causality: as previously mentioned in the section “Introduction”; elderly individuals with greater AD pathologies may participate in less CA (Jack et al., 2013; Villemagne et al., 2013). This explanation was further supported by the sensitivity analysis conducted for the CN subgroup. In the CN subgroup, no trend level association was observed between CA_late_ and AD-related brain changes, which is consistent with previous reports (Landau et al., 2012; Wirth et al., 2014; Gidicsin et al., 2015).

In a sensitivity analysis, we controlled for the effect of CA_early_ as well as education when analyzing the relationship between CA_mid_ or CA_late_ and global Aβ deposition and AD-CMglu, because CA_mid_ and CA_late_ were correlated with CA_early_. Controlling for CA_early_ did not change the results, indicating that the negative findings for the relationship of CA_mid_ or CA_late_ with AD-related brain change were significant, regardless of the influence of CA_early_.

There are several limitations to our study. First, although we used well-validated and reliable questionnaires, retrospective measurements of CA may have a recall bias. Current depression and memory impairment have the potential to affect retrospective measurements based on subjective recall. To mitigate the potential risk, we conducted two sensitivity analyses. We controlled for current depression using the GDS score. This did not change the overall results of our study. Furthermore, the same analyses conducted for the CN group revealed potential associations between CA_early_ and both AD-CMglu and AD-ND positivity, although not statistically significant. Future long-term prospective studies are required to confirm our findings. Second, as for AD-related neurodegeneration, we measured cerebral glucose metabolism by FDG-PET. Although we defined AD-CMglu or AD-ND positivity by applying AD-signature regions showing typical AD-pattern hypometabolism, glucose metabolism may be influenced by non-AD pathologies, such as vascular pathology and non-AD degenerative conditions (Kato et al., 2016). Tau-PET imaging (Saint-Aubert et al., 2016) or CSF phosphorylated tau measurements (Blennow and Hampel, 2003) may provide information to address this issue. Third, we did not consider the influence of potential confounding factors, which may affect the *in vivo* AD pathologies, such as physical activity (Shah et al., 2014), social interaction (Bennett et al., 2006), diet (Berti et al., 2015), oxidative stress (Markesbery, 1997), and various physical conditions, including hypertension, diabetes, obesity, and other chronic illnesses (Chui et al., 2012), although we excluded individuals with serious medical or neurological disorders that may influence mental functioning.

## Conclusion

Our results support that CA in early life is probably protective against late-life AD-related neurodegeneration, independently of cerebral Aβ pathology. In contrast, CA in midlife and late life appears to have no or limited association with AD-related brain changes, including amyloid pathology and neurodegeneration. With respect to prevention of dementia and cognitive impairment in late life, a cognitively active lifestyle in childhood and early adulthood needs to be more emphasized.

## Ethics Statement

This study protocol was approved by the Institutional Review Boards of Seoul National University Hospital (C-1401-027-547) and SNU-SMG Boramae Center, Seoul, South Korea (26-2015-60), and was conducted in accordance with the recommendations of the current version of the Declaration of Helsinki. All subjects provided written informed consents.

## Author Contributions

KK and DL designed the study, acquired and interpreted the data, and were major contributors to the writing of the manuscript and critically revising the manuscript for intellectual content. MB, DY, JL, and CK acquired and analyzed the data and helped to draft the manuscript. KK and DY analyzed the imaging data. DL served as the principal investigator and supervised the study. All authors read and approved the final manuscript.

## Conflict of Interest Statement

The authors declare that the research was conducted in the absence of any commercial or financial relationships that could be construed as a potential conflict of interest.
