# Supplementary material for: Early-Life Cognitive Activity Is Related to Reduced Neurodegeneration in Alzheimer Signature Regions in Late Life
Source: Front Aging Neurosci. 2018 Mar 22;10:70. doi: 10.3389/fnagi.2018.00070 (PMC5875443; doi:10.3389/fnagi.2018.00070)
Supplement: Supplementary file 1 [file Table_1.docx]

***Supplementary Material***

**Early-life cognitive activity relates to reduced Alzheimer neurodegeneration in late-life**

**Kang Ko, MD^1,2^, Min Soo Byun, MD, PhD^3^, Dahyun Yi, PhD^3^, Jun Ho Lee, MD^1,4^, Chan Hyung Kim, MD, PhD^2,5^, Dong Young Lee, MD, PhD^1,3,4,*^ and for the KBASE research group^#^**

^1^Department of Neuropsychiatry, Seoul National University Hospital, Seoul, Republic of Korea

^2^Department of Psychiatry, Yonsei University College of Medicine, Seoul, Republic of Korea

^3^Institute of Human Behavioral Medicine, Medical Research Center, Seoul National University, Seoul, Republic of Korea

^4^Department of Psychiatry, Seoul National University College of Medicine, Seoul, Republic of Korea

^5^Institute of Behavioral Science in Medicine, Yonsei University College of Medicine, Seoul, Republic of Korea

^#^Information of the KBASE Research Group is provided in the online supplemental material.

*** Correspondance: Dong Young Lee,** selfpsy@snu.ac.kr

**Appendix e-1.** Image acquisition and preprocessing

**Appendix e-2.** Receiver operating characteristic (ROC) curve analyses to define threshold for neurodegeneration biomarkers abnormality

**Appendix e-3.** Information of the KBASE research group

**Table e-1.** Interaction between early life cognitive activity and APOE4 carrier status on AD-CMglu

**Table e-2.** Association of cognitive activities in each lifetime period with global Aβ deposition and AD-CMglu (additionally adjusted for GDS)

**Table e-3.** Association of cognitive activities in each lifetime period with Aβ and AD-ND positivity (additionally adjusted for GDS)

**Table e-4.** Association of cognitive activities in each lifetime period with global Aβ deposition and AD-CMglu (within CN elderly, additionally adjusted for GDS)

**Table e-5.** Association of cognitive activities in each lifetime period with Aβ and AD-ND positivity (within CN elderly, additionally adjusted for GDS)

**Table e-6.** Association of CA_mid_ and CA_late_ with global Aβ deposition and AD-CMglu (additionally adjusted for CA_early_)

**Table e-7.** Association of CA_mid_ and CA_late_ with Aβ and AD-ND positivity (additionally adjusted for CA_early_)

**Appendix e-1. Image acquisition and preprocessing**

**1. [^11^C]Pittsburg compound B (PiB) – positron emission tomography (PET) image acquisition and preprocessing**

Participants underwent simultaneous three-dimensional (3D) PiB-PET and 3D T1-weighted magenetic resonance (MR) imaging using a 3.0T Biograph mMR scanner (PET-MR scanner; Siemens, Washington DC, USA) according to the manufacturer’s approved guidelines. After intravenous administration of 555 MBq of [^11^C]PiB (range, 450-610 MBq), a 30-minute emission scan was obtained 40 minutes after injection. The PiB-PET data collected in list mode were processed for routine corrections such as uniformity, UTE-based attenuation, and decay corrections, and were reconstructed into a 256 x 256 image matrix using iterative methods (6 iterations with 21 subsets).

The following image preprocessing steps were performed using Statistical Parametric Mapping 8 (SPM8) implemented in Matlab 2014a (Mathworks, Natick, MA, USA). Static PiB-PET images were co-registered to individual T1 structural images and transformation parameters for the spatial normalization of individual T1 images to a standard Montreal Neurological Institute (MNI) template were calculated. Using IBASPM software, we used the inverse transformation parameters to transform coordinates from the automatic anatomic labeling (AAL) 116 atlas into an individual space for each subject (a resampling voxel size = 1 x 0.98 x 0.98 mm), and the non-gray matter portions of the atlas were individually masked using the cerebral gray matter segment image from each subject.

The mean regional ^11^C-PiB uptake values from cerebral regions were extracted using the individual AAL116 atlas from T1-coregistered PiB-PET images. Cerebellar gray matter was used as the reference region for quantitative normalization of cerebral PiB uptake values due to its relatively low Aβ deposition. To measure PiB uptake in the cerebellar gray matter regions, a probabilistic cerebellar atlas (Institute of Cognitive Neuroscience, UCL; Cognitive Neuroscience Laboratory, Royal Holloway) was transformed into individual space in the same manner as described above. Of the 28 anatomical structural regions in the cerebellar atlas, all cerebellar lobular regions except the vermis were included to extract the mean cerebellar uptake values.

The AAL algorithm and a region combining method were applied to determine regions of interests (ROIs) to characterize the ^11^C-PiB retention level in the frontal, lateral parietal, posterior cingulate-precuneus, and lateral temporal regions. The standardized uptake value ratio (SUVR) values for each ROI were calculated by dividing the mean PiB uptake value for all voxels within each ROI by the mean cerebellar PiB uptake value in the same image. A global cortical ROI consisting of the four ROIs was also defined, and a global Aβ retention value was generated by dividing the mean value for all voxels of the global cortical ROI by the mean cerebellar uptake value in the same image.

**2. [^18^F]Fluorodeoxyglucose (FDG)-PET image acquisition and preprocessing**

The participants fasted for at least 6 hours and rested in a waiting room for 40 minutes prior to the scans after intravenous administration of 0.1 mCi/Kg of [^18^F]FDG radioligands. The PET data collected in list mode (5 minutes x 4 frames) were processed for routine corrections such as uniformity, UTE-based attenuation, and decay corrections. After inspecting the data for any significant head movements, we reconstructed them into a 20-minute summed image using iterative methods (6 iterations with 21 subsets).

The following image processing steps were performed using SPM12 (http://www.fil.ion.ucl.ac.uk/spm) implemented in Matlab 2014a (Mathworks, Natick, MA, USA). First, static FDG-PET images were co-registered to individual T1 structural images, and transformation parameters for the spatial normalization of individual T1 images to a standard MNI template were calculated and used to spatially normalize the PET images to the MNI template. After smoothing the spatially normalized FDG-PET images with a 12-mm Gaussian filter, intensity normalization was performed using the pons as the reference region.

**Appendix e-2. Receiver operating characteristic (ROC) curve analyses to define threshold for neurodegeneration biomarkers abnormality**

ROC curve analyses was performed to determine the optimal threshold about neurodegeneration positivity that can distinguish the AD dementia from the cognitively normal (CN) elderly individuals.

For these ROC analyses, data from AD dementia subjects and CN subjects in KBASE cohort were used. Inclusion criteria for the AD dementia group were as follows: (a) aged 55 – 90 years (inclusive), (b) Clinical Dementia Rating (CDR) score 0.5 or 1, and (c) Probable AD dementia according to the National Institute of Aging and the Alzheimer's Association (NIA-AA) diagnostic criteria for AD. Inclusion criteria for CN and the exclusion criteria for both groups are described in the manuscript.

To set the threshold of the SUVR value in the AD-signature FDG ROI that can distinguish AD dementia from CN, data from 58 AD dementia subjects (mean age: 72.9 ± 8.1 years; female/male: 41/17; global CDR 0.8 ± 0.2) and 260 CN subjects (mean age: 68.7 ± 8.0 years; female/male: 134/126; global CDR 0.0 ± 0.0) were used. Using CN subjects as the reference group, we set the optimal cut-off point of SUVR in the AD-signature FDG ROI based on the Youden index at 1.386 (sensitivity 91.4%).

**Appendix e-3. Information of the KBASE Research Group**

**1. Participating investigators**

Dong Young Lee, MD, PhD (Seoul National University, Principal Investigator); Min Soo Byun, MD, PhD (Seoul National University, Core PI Clinical & Executive); Dahyun Yi, PhD (Seoul National University, Core PI Neuropsychology); Yu Kyeong Kim, MD, PhD (SMG-SNU Boramae Medical Center, Core PI PET); Chul-Ho Sohn, MD, PhD (Seoul National University, Core PI MRI); Inhee Mook-Jung, PhD (Seoul National University, Core PI Biomarker); Murim Choi, PhD (Seoul National University, Core PI Genetics); Yu Jin Lee, MD, PhD (Seoul National University, Core PI Sleep), Seokyung Hahn, PhD (Seoul National University, Core PI Biostatistics); Hyun Jung Kim, MD (Changsan Convalescent Hospital, co-investigator); Mun Young Chang, MD (Chung-Ang University College of Medicine, co-investigator); Seung Hoon Lee, MD (Daerim St. Mary's Hospital, co-investigator); Jee Wook Kim, MD, PhD (Hallym University Dongtan Sacred Heart Hospital, co-investigator); Jong-Min Lee, PhD (Hanyang University, co-investigator); Dong Woo Lee, MD, PhD (Inje University Snaggye Paik Hospital, co-investigator); Bo Kyung Sohn, MD (Inje University Snaggye Paik Hospital, co-investigator); Seok Woo Moon, MD, PhD (Konkuk University Chungju Hospital, co-investigator); Man Ho Choi, PhD (Korea Institute of Science and Technology, co-investigator); Sang-Won Lee, PhD (Korea University, co-investigator); Hyewon Baek, MD (Kyunggi Provincial Hospital for the Elderly, co-investigator); Na Young Han, MD (National Research Center for Dementia, co-investigator); Jong-Won Kim, MD, PhD (Samsung Medical Center, co-investigator); Seung-Ho Ryu, MD, PhD (School of Medicine Konkuk University, co-investigator); Shin Gyeom Kim, MD, PhD (Soonchunhyang University Hospital Bucheon, co-investigator); Sun-Ho Han, PhD (Seoul National University, co-investigator); Jae Sung Lee, PhD (Seoul National University, co-investigator); Yun-Sang Lee, PhD (Seoul National University, co-investigator); Jong Inn Woo, MD, PhD (Seoul National University, co-investigator); Sang Eun Kim, MD, PhD (Seoul National University Bundang Hospital, co-investigator); Byung Chul Lee, PhD (Seoul National University Bundang Hospital, co-investigator); Gi Jeong Cheon, MD, PhD (Seoul National University Hospital, co-investigator); Koung Mi Kang, MD (Seoul National University Hospital, co-investigator); Jee-Eun Park, MD, PhD; (Seoul National University Hospital, co-investigator); Hyeong Gon Yu, MD, PhD (Seoul National University Hospital, co-investigator); Jun-Young Lee, MD, PhD (SMG-SNU Boramae Medical Center, co-investigator); Hyo Jung Choi, MD (SMG-SNU Boramae Medical Center, co-investigator); Young Min Choe, MD (University of Ulsan College of Medicine, Ulsan University Hospital, co-investigator); Woonhyung Ghim, MD (Seoul National University Hospital, research fellow); So Yeon Jeon, MD (Seoul National University Hospital, research fellow); Woo Jin Kim, MD, PhD (Seoul National University Hospital, research fellow); Kang Ko, MD (Seoul National University Hospital, research fellow); Jun Ho Lee, MD (Seoul National University Hospital, research fellow); Kyoungjin Chu (Seoul National University Hospital, psychologist); Hyunwoong Ko (Seoul National University Hospital, psychologist); Younghwa Lee (Seoul National University Hospital, psychologist); Donghwi Hwang (Seoul National University, image analyst); Seugn Kwan Kang (Seoul National University, image analyst); Seong A Shin (Seoul National University, image analyst); Jeong Yeon Hwang, MD (Seoul National University, data analyst); Jong-Chan Park (Seoul National University, data analyst); Jong-Ho Park (Samsung Medical Center, genetic data analyst); Jieun Seo (Seoul National University, genetic data analyst); Mi Ae Han (Seoul National University Hospital, research coordinator); Eun A Jo (Seoul National University Hospital, research coordinator); Gi Jung Jung (Seoul National University Hospital, research coordinator); Jin Hee Keum (Seoul National University Hospital, research coordinator); Mi Sun Kim (SMG-SNU Boramae Medical Center, research coordinator); Min Jeong Kim (Seoul National University Hospital, research coordinator); Han Na Lee (Seoul National University Hospital, research coordinator); Bo Eun Park (Seoul National University Hospital, research coordinator); Ji Sun Shin (Seoul National University Hospital, research coordinator); Yun Jung Hwang (Seoul National University Hospital, researcher); Joon Hyung Jung, MD (Seoul National University Hospital, researcher); Kiyoung Sung, MD (Seoul National University Hospital, researcher); Eun Hye Kim (Seoul National University, research assistant); Han Byul Choi (National Research Center for Dementia, administrative staff)

**2. Partner Organization**

Dongjak-gu Center for Dementia

Jongno-gu Center for Dementia

Ministry of Science, ICT and Future planning

National Research Center for Dementia

National Research Foundation of Korea

Seoul Metropolitan Center for Dementia

Seoul National University

Seoul National University Bundang Hospital

Seoul National University Hospital

SMG-SNU Boramae Medical Center

The Korean Association for Dementia

**Table e-1. Interaction between CA_early_ and APOE4 carrier status on AD-CMglu**

|  | **B** | **SE** | **Beta** | ***P*** |
| --- | --- | --- | --- | --- |
| CA_early_ | 0.043 | 0.015 | 0.221 | .005 |
| APOE4 | 0.057 | 0.057 | 0.182 | .32 |
| CA_early_ X APOE4 | -0.030 | 0.025 | -0.219 | .24 |

Key: CA_early_, early-life cognitive activity; APOE4, Apolipoprotein E ε4; AD-CMglu, Alzheimer’s disease signature region cerebral glucose metabolism.

Note: The results of general linear model with age, gender, and education as covariates are presented.

**Table e-2. Association of CAs in each lifetime period with global Aβ deposition and AD-CMglu (additionally adjusted for GDS)**

|  | **B** | **S.E** | **Beta** | ***P*** | ***P_B_*** |
| --- | --- | --- | --- | --- | --- |
| **Global Aβ deposition** | | | | | |
| Early life | -0.024 | 0.036 | -0.046 | .51 | >.99 |
| Midlife | -0.004 | 0.032 | -0.008 | .91 | >.99 |
| Late life | -0.068 | 0.034 | -0.134 | .05 | .14 |
| **AD-CMglu** | | | | |  |
| Early life | 0.034 | 0.014 | 0.175 | .01 | .04 |
| Midlife | 0.008 | 0.012 | 0.051 | .49 | >.99 |
| Late life | 0.026 | 0.013 | 0.143 | .04 | .12 |

Key: Aβ, amyloid-beta; AD-CMglu, Alzheimer’s disease signature region cerebral glucose metabolism.

Note: The results of independent multiple linear regression model with age, gender, education, apolipoprotein E ε4 and GDS as covariates are presented.

*P_B_*: *P*-value corrected by Bonferroni’s method.

**Table e-3. Association of CAs in each lifetime period with Aβ and AD-ND positivity (additionally adjusted for GDS)**

|  | **Adjusted OR** | **95% CI** | ***P*** | ***P_B_*** |
| --- | --- | --- | --- | --- |
| **Aβ positivity** |  |  |  |  |
| Early life | 0.879 | 0.521-1.483 | .63 | >.99 |
| Midlife | 1.007 | 0.630-1.610 | .98 | >.99 |
| Late life | 0.841 | 0.514-1.378 | .49 | >.99 |
| **AD-ND positivity** | | | |  |
| Early life | 0.663 | 0.436-1.008 | .05 | .16 |
| Midlife | 0.814 | 0.564-1.176 | .27 | .85 |
| Late life | 0.736 | 0.496-1.092 | .13 | .38 |

Key: OR, odds ratio; CI, confidence interval; Aβ, amyloid-beta; AD-ND, Alzheimer’s disease signature region neurodegeneration.

Note: The results of independent multiple logistic regression model with age, gender, education, apolipoprotein E ε4 and GDS as covariates are presented.

*P_B_*: *P*-value corrected by Bonferroni’s method.

**Table e-4.** **Association of CAs in each lifetime period with global Aβ deposition and AD-CMglu (within CN elderly, additionally adjusted for GDS)**

|  | **B** | **S.E** | **β** | ***P*** | ***P_B_*** |
| --- | --- | --- | --- | --- | --- |
| **Global Aβ deposition** |  |  |  |  |  |
| Early life | -0.005 | 0.028 | -0.014 | .86 | >.99 |
| Midlife | 0.018 | 0.026 | 0.059 | .49 | >.99 |
| Late life | 0.002 | 0.027 | 0.004 | .96 | >.99 |
| **AD-CMglu** | | | | | |
| Early life | 0.025 | 0.013 | 0.154 | .05 | .16 |
| Midlife | 0.006 | 0.012 | 0.044 | .61 | >.99 |
| Late life | 0.008 | 0.013 | 0.051 | .53 | >.99 |

Key: Aβ, amyloid-beta; AD-CMglu, Alzheimer’s disease signature region cerebral glucose metabolism.

Note: The results of independent multiple linear regression model with age, gender, education, apolipoprotein E ε4 and GDS as covariates are presented.

*P_B_*: *P*-value corrected by Bonferroni’s method.

**Table e-5. Association of CAs in each lifetime period with Aβ and AD-ND positivity (within CN elderly, additionally adjusted for GDS)**

|  | **Adjusted OR** | **95% CI** | ***P*** | ***P_B_*** |
| --- | --- | --- | --- | --- |
| **Aβ positivity** |  |  |  |  |
| Early life | 0.931 | 0.437-1.985 | .85 | >.99 |
| Midlife | 1.381 | 0.667-2.858 | .38 | >.99 |
| Late life | 1.324 | 0.641-2.736 | .45 | >.99 |
| **AD-ND positivity** | | | |  |
| Early life | 0.636 | 0.396-1.023 | .06 | .19 |
| Midlife | 0.724 | 0.469-1.116 | .14 | .43 |
| Late life | 0.838 | 0.531-1.323 | .45 | >.99 |

Key: OR, odds ratio; CI, confidence interval; Aβ, amyloid-beta; AD-ND, Alzheimer’s disease signature region neurodegeneration.

Note: The results of independent multiple logistic regression model with age, gender, education, apolipoprotein E ε4 and GDS as covariates are presented.

*P_B_*: *P*-value corrected by Bonferroni’s method.

**Table e-6. Association of CA_mid_ and CA_late_ with global Aβ deposition and AD-CMglu (additionally adjusted for CA_early_)**

|  | **B** | **S.E** | **Beta** | ***P*** | ***P_B_*** |
| --- | --- | --- | --- | --- | --- |
| **Global Aβ deposition** | | | | | |
| Midlife | 0.005 | 0.037 | -0.008 | .90 | >.99 |
| Late life | -0.072 | 0.036 | -0.143 | .05 | .14 |
| **AD-CMglu** | | | | |  |
| Midlife | -0.008 | 0.014 | -0.046 | .58 | >.99 |
| Late life | 0.018 | 0.013 | 0.098 | .18 | .54 |

Key: Aβ, amyloid-beta; AD-CMglu, Alzheimer’s disease signature region cerebral glucose metabolism.

Note: The results of independent multiple linear regression model with age, gender, education, apolipoprotein E ε4 and CA_early_ as covariates are presented.

*P_B_*: *P*-value corrected by Bonferroni’s method.

**Table e-7. Association of CA_mid_ and CA_late_ with Aβ and AD-ND positivity (additionally adjusted for CA_early_)**

|  | **Adjusted OR** | **95% CI** | ***P*** | ***P_B_*** |
| --- | --- | --- | --- | --- |
| **Aβ positivity** |  |  |  |  |
| Midlife | 1.020 | 0.601-1.733 | .94 | >.99 |
| Late life | 0.812 | 0.480-1.374 | .44 | >.99 |
| **AD-ND positivity** | | | |  |
| Midlife | 0.944 | 0.619-1.439 | .79 | >.99 |
| Late life | 0.803 | 0.529-1.219 | .30 | .91 |

Key: OR, odds ratio; CI, confidence interval; Aβ, amyloid-beta; AD-ND, Alzheimer’s disease signature region neurodegeneration.

Note: The results of independent multiple logistic regression model with age, gender, education, apolipoprotein E ε4 and CA_early_ as covariates are presented.

*P_B_*: *P*-value corrected by Bonferroni’s method.
